# PReS13-SPK-1592: Pediatric aspects of antiphospholipid syndrome

**DOI:** 10.1186/1546-0096-11-S2-I13

**Published:** 2013-12-05

**Authors:** T Avcin

**Affiliations:** 1 Department of Allergology, Rheumatology and Clinical Immunology, Children's Hospital, University Medical Center Ljubljana, Ljubljana, Slovenia

## Introduction

The antiphospholipid antibody syndrome (APS) is a multisystemic autoimmune disease characterized by thromboembolic events, pregnancy morbidity, hematologic, dermatologic, neurologic and other manifestations in the presence of elevated titers of antiphospholipid antibodies (aPL). In recent years, APS has been increasingly recognized in various pediatric autoimmune and nonautoimmune diseases, but the relatively low prevalence and heterogeneity of APS in childhood made it very difficult to study in a systematic way.

## Objective

To evaluate the prevalence of thrombotic and nonthrombotic clinical manifestations in children with positive aPL and determine the long-term outcome of children with APS.

## Methods

A retrospective study with longitudinal follow-up of an unselected group of children who tested positive for at least one aPL subtype (aCL, anti-β2GPI and/or LA) was conducted for assessment of the prevalence of clinical features associated with aPL in children. Testing for aPL was requested by treating physicians given the clinical suspicion of aPL-related clinical manifestations. Data from the European registry extended internationally of pediatric patients with APS (Ped-APS Registry) were used for assessment of the long-term outcome of children with APS. To be eligible for enrollment the patient must meet the preliminary criteria for the classification of pediatric APS and the onset of APS must have occurred prior to the patient's 18th birthday.

## Results

A total of 159 patients who tested positive for at least one aPL subtype (aCL, anti-β2GPI and/or LA) at the time of or within the first three months after the disease presentation were enrolled. There were 98 (62%) females and 61 (38%) males with mean age at disease presentation 11.4 years (range: 1-18 years). Mean follow-up period was 6.2 years. During this period 25 (16%) patients had thrombotic event (16 venous and 9 arterial), 48 (30%) patients developed hematological manifestations, 25 (16%) patients nonthrombotic neurological manifestations, 19 (12%) patients skin manifestations and 5 (3%) patients cardiac valve disease. Two out of 25 (8%) patients with thrombosis had recurrent thrombotic event. Underlying systemic autoimmune disease was identified in 55 (35%) of patients.

The clinical characteristics and outcome was compared with 140 children with APS included in the Ped-APS Registry. Venous thrombosis occurred in 86 (61%), arterial thrombosis in 43 (31%), small vessel thrombosis in 7 (5%) and mixed arterial and venous thrombosis in 4 (3%) patients. Associated non-thrombotic clinical manifestations included hematological manifestations (39%), skin disorders (19%) and non-thrombotic neurological manifestations (16%). Mean follow-up time from the time of APS diagnosis was 6.1 years. Recurrent thrombosis was observed in 19% of pediatric patients with initial venous thrombosis and 21% of patients with initial arterial thrombosis. Sixty-eight (49%) patients included in the Ped-APS Registry had underlying autoimmune disease.

## Discussion

Nonthrombotic clinical manifestations were more frequent than thrombotic events (16%) in an unselected group of children with positive aPL. Children with APS have significantly higher thrombosis recurrence rates as compared with adult APS patients.

## Disclosure of interest

None declared.

